# Identifying Thyroid Carcinoma-Related Genes by Integrating GWAS and eQTL Data

**DOI:** 10.3389/fcell.2021.645275

**Published:** 2021-02-04

**Authors:** Fei Shen, Xiaoxiong Gan, Ruiying Zhong, Jianhua Feng, Zhen Chen, Mengli Guo, Yayi Li, Zhaofeng Wu, Wensong Cai, Bo Xu

**Affiliations:** ^1^Department of Thyroid Surgery, Guangzhou First People’s Hospital, School of Medicine, South China University of Technology, Guangzhou, China; ^2^Department of Thyroid Surgery, Guangzhou First People’s Hospital, Guangzhou Medical University, Guangzhou, China; ^3^Department of Hepatobiliary, Pancreatic and Splenic Surgery, Guangzhou First People’s Hospital, School of Medicine, South China University of Technology, Guangzhou, China

**Keywords:** thyroid carcinoma, rs1912998, IGFALS, HAGH, GWAS, eQTL

## Abstract

Thyroid carcinoma (TC) is the most common endocrine malignancy. The incidence rate of thyroid cancer has increased rapidly in recent years. The occurrence and development of thyroid cancers are highly related to the massive genetic and epigenetic changes. Therefore, it is essential to explore the mechanism of thyroid cancer pathogenesis. Genome-Wide Association Studies (GWAS) have been widely used in various diseases. Researchers have found multiple single nucleotide polymorphisms (SNPs) are significantly related to TC. However, the biological mechanism of these SNPs is still unknown. In this paper, we used one GWAS dataset and two eQTL datasets, and integrated GWAS with expression quantitative trait loci (eQTL) in both thyroid and blood to explore the mechanism of mutations and causal genes of thyroid cancer. Finally, we found rs1912998 regulates the expression of IGFALS (*P* = 1.70E-06) and HAGH (*P* = 5.08E-07) in thyroid, which is significantly related to thyroid cancer. In addition, KEGG shows that these genes participate in multiple thyroid cancer-related pathways.

## Introduction

Thyroid carcinoma (TC) is one of the most common endocrine tumors (Cabanillas et al., 2016). The incidence rate has increased year by year in recent 30 years, driven largely by increases in Differentiated TC. At present, the research and treatment of TC are still unclear. Ionizing radiation is the only known carcinogenic mechanism, while other causes are still unclear (Albi et al., 2017). Differentiated TC is the most frequent subtype, and the main treatment methods of differentiated TC include surgical treatment (Asimakopoulos and Nixon, 2018), radioiodine therapy (Klain et al., 2002) and thyroid-stimulating hormone (TSH) suppression treatment (Haugen et al., 2016), while the treatment of poorly differentiated thyroid cancer (PDTC) is still very limited (Ibrahimpasic et al., 2019). With the development of sequencing technology and the decrease of cost, research on TC is progressing at the genetic level, it has been found that many genes are related to the occurrence, development, and prognosis of TC, which provides a new direction for early diagnosis, prognosis judgment and targeted treatment of TC.

BRAF gene plays an important role in the development of TC, especially in papillary thyroid cancer (PTC) (Pusztaszeri et al., 2015). BRAF gene mutations are most common in PTC, and less in other pathological types (Chen et al., 2018). Multiple biological experiments in mice and humans have found that BRAF mutation is significantly associated with the prognosis of PTC (Nakamura et al., 2005; Zou et al., 2009; Ohori et al., 2013). RET mutations are closely associated with hereditary myeloid carcinoma-related diseases, such as familial thyroid myeloid carcinoma, multiple endocrine adenomatosis type 2A, multiple endocrine adenomatosis type 2B, etc. (Gandhi et al., 2010; Accardo et al., 2017). It has also been suggested that RET gene plays an important role in evaluating the need for preventive thyroidectomy and the selection of surgical operation (Cohen and Moley, 2003; Hirsch et al., 2018). However, studies have found that the rate of RET gene mutations in Chinese PTC patients is very low, which means the relationship between RET and TC still needs further study (Wang et al., 2008). RAS mutations also occur in thyroid cancers, which frequently occur in follicular thyroid cancer and follicular variant papillary thyroid cancer (Garcia-Rendueles et al., 2015; Cabanillas et al., 2016). Other wide known TC-related genes, such as TP53 (Wang et al., 2014), PAX8-PPAR (Boos et al., 2013), TERT (Liu and Xing, 2016), PTEN (Puxeddu et al., 2005), and KRAS (Al-Salam et al., 2020), etc., are all found by complicated biological experiments. This leads to a high cost in time and money.

Using the computational method to handle complex networks provides a new way to study disease-related genes (Zhao et al., 2020a,b). The most commonly used network is the gene regulatory network which is a directed graph. Genes are the nodes of the network, and the regulatory relationship between genes is used to connect nodes and form directed edges (Zhao et al., 2020c). A large number of computational methods based on complex networks have been studied. Most of these methods identify important genes by measuring the centrality of network nodes, and the degree centrality method is representative of this kind of methods. The degree of a node is the number of nodes directly connected with it. It is generally believed that the degree of gene is directly proportional to the importance of gene. According to the importance of genes, we can sort the relationship between genes and diseases in the network.

Genome-Wide Association Studies (GWAS) have been widely used in various diseases. Rogounovitch et al. (2015) conducted a GWAS study using 507 sporadic PTC patients and 2,766 control subjects in the Japanese population. Jones et al. (2012) compared 781 PTC patients with 6,122 health controls in England population to find TC-related SNPs. Penna-Martinez et al. (2014) categorized rs944289 in 243 patients with differentiated TC and 270 healthy individuals in the German population, and the results indicated that rs944289 may play a minor role in the occurrence of differentiated thyroid cancer in the German population. Wang et al. (2013) selected 845 patients with PTC, 503 patients with thyroid benign Node (TBN), and 1,005 healthy controls in east China to conduct GWAS, and found that four candidate loci, rs965513 (9q22.33), rs944289 (14q13.3), rs966423 (2q35), and rs2439302 (8p12), identified for PTC risk in a Chinese population. Wei et al. (2015) collected anticoagulant blood samples from 1,237 thyroid tumor patients and 760 healthy people of Chinese Han nationality, and collected and compared clinic-pathological data. Although these GWAS studies revealed multiple TC-related SNPs, the biological mechanism between TC and these SNPs is still unknown.

Expression quantitative trait loci (eQTL) refers to regions on chromosomes that can specifically regulate the expression level of mRNA/protein, and the expression level of mRNA/protein is proportional to the quantitative traits (Doss et al., 2005). eQTL can be divided into *cis-*eQTL and *trans-*eQTL. *Cis-*eQTL is that the eQTL of a gene is located in the genomic region of the gene, which indicates that the change of mRNA level may be caused by the difference of the gene itself. Overall, eQTL describes the ability of SNP to regulate gene expression.

At present, a considerable number of studies have taken eQTL as a very effective tool for in-depth interpretation of GWAS results (Yin et al., 2014; Zhao et al., 2020d, 2021). With the increase of sample size, the problem of low statistical efficiency caused by the small sample size in the past has gradually improved. Recently, studies on eQTL have gradually begun to be carried out in several major tissues of human body, and it has been found that many eQTLs are tissue-specific, that is, some SNPs only work in specific tissues or cell types (Van Nas et al., 2010; Simon et al., 2016; Battle et al., 2017). Therefore, the thyroid and peripheral blood of eQTL are needed to study TC-related genes.

In this study, a mendelian randomization-based method was used to integrate GWAS and eQTL data, which reveals the biological mechanism of significant SNPs and causal genes of TC.

## Materials and Methods

### Dataset

In this study, we used one GWAS dataset and two eQTL datasets. Rashkin et al. (2020) undertook GWAS and conducted a comprehensive assessment of the heritability and pleiotropy of 18 cancer types in two large, population-based cohorts: the United Kingdom Biobank (408,786 European ancestry individuals; 48,961 cancer cases) and the Kaiser Permanente Genetic Epidemiology Research on Adult Health and Aging cohorts (66,526 European ancestry individuals; 16,001 cancer cases).

**FIGURE 1:**
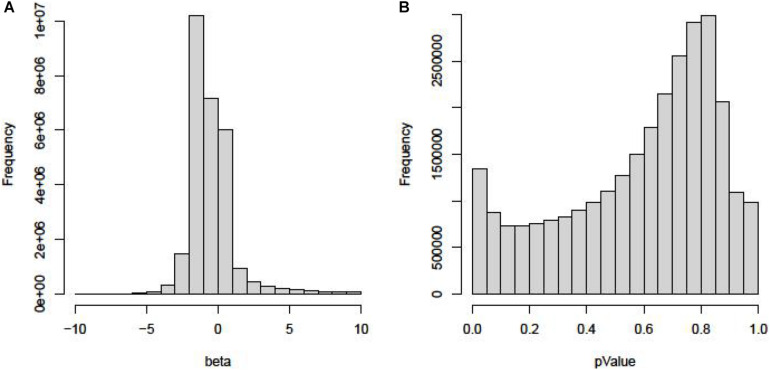
The “Beta” and “*P*-value” of all SNPs in the GWAS dataset. **(A)** Histogram of Beta in GWAS. **(B)** Histogram of *P* value in GWAS.

The “Beta” and “*P* value” of all SNPs in the GWAS dataset are shown in Figures 1A,B.

As we can see in Figure 1A, most of the beta are between −1 and −2. Few SNPs have high beta values, which mean that these mutations pose a high risk of TC. In Figure 1B, the *P* value of 99% SNPs is higher than 0.05.

**FIGURE 2:**
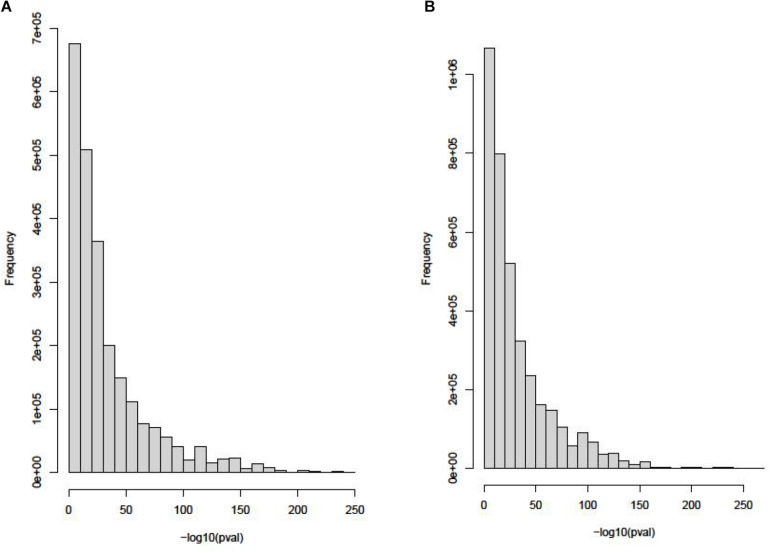
The distributions of blood tissue and thyroid tissue in the eQTL data. **(A)** Histogram of *P* value in eQTL of blood tissue. **(B)** Histogram of *P* value in eQTL of thyroid tissue.

The eQTL data of blood tissue is downloaded from Genotype-Tissue Expression (GTEx) (GTEx Consortium, 2013). Using 12,360 gene expression probes, this dataset includes 1,272,372 eQTL loci. eQTL data of thyroid tissue is downloaded from GTEx too. Using 17,664 gene expression probes, this dataset includes 1,757,598 eQTL loci. The distributions of these two datasets are shown in Figure 2.

### Summary Data-Based Mendelian Randomization

Potential and unmeasurable confounding factors, such as growth environment, economic factors, behavior, and so on, lead to great challenges in inferring the causal relationship between genes and TC. The allele Randomization rate in Mendelian genetic law is an important theoretical support of the Mendelian Randomization (MR) method. If the phenotype is determined by genotype, a genotype is related to the disease by controlling phenotype. Therefore, the genotype can be used as an instrumental variable to infer the causal relationship between phenotype and disease.

Zhu et al. (2016) developed a summary data-based Mendelian Randomization (SMR) method to test the associations between gene expression and five complex traits. Then, SMR has been widely used in various diseases, such as Alzheimer’s disease (AD) (Zhao et al., 2019, 2020e), Bone Mineral Density (BMD) (Meng et al., 2018), Amyotrophic Lateral Sclerosis (ALS) (Du et al., 2018), etc.

Let x be exposure factors (Gene expression), y be outcome variables (phenotype), z be instrumental variable (SNP). *b*_*xy*_ is defined as the effect of x on y.

(1) $$b_{xy}=b_{zy}/b_{zx}$$

The variance of the association between gene and TC should be:

(2) $$var\left(b_{xy}^{\prime }\right)=\left[var\left(y\right)\left(1-R_{xy}^{2}\right)\right]/\left[nvar\left(x\right)R_{zx}^{2}\right]$$

$b_{xy}^{\prime }$ is the estimated value of *b*_*xy*_. $R_{xy}^{2}$ is the variance that gene expression regresses to the TC. $R_{zx}^{2}$ is the variance that SNP regresses to the gene expression. n is the sample size.

Therefore, we can define *T*_*SMR*_ to test the significance of b_*xy.*_

(3) $$T_{SMR}=b_{xy}^{\prime ,2}/var\left(b_{xy}^{\prime }\right)$$

where $b_{zx}^{\prime }=z_{zx}S_{zx}$, $S_{zx}=1\sqrt{2p\left(1-p\right)\left(n+z_{zx}^{2}\right)}$, p is the Allele frequency.

Since *T*_*SMR*_ obeys the chi-square distribution with degree of freedom of 1, we can obtain a *P* value for each SNP.

**FIGURE 3:**
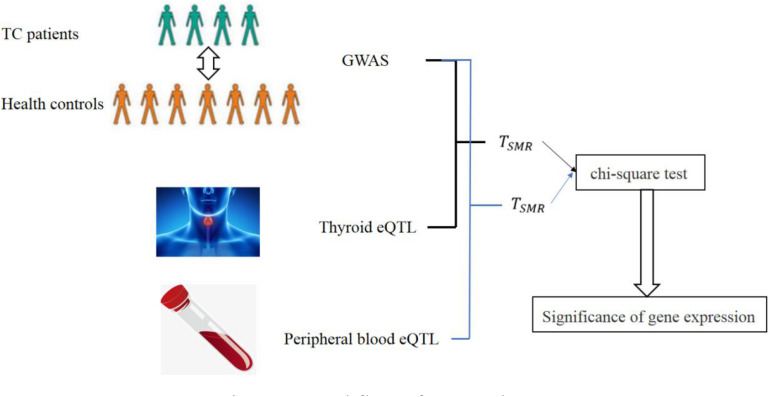
Workflow of our work.

The workflow of our work is as following:

As shown in Figure 3, we can obtain the statistic *T*_*SMR*_ from GWAS and eQTL. Since we used eQTL datasets from two tissues, we totally obtained two *T*_*SMR*_. We test *T*_*SMR*_ by chi-square test to find casual genes of TC.

## Results

### Results of SMR Test

Since 12,360 probes in the peripheral blood eQTL data set labeled all SNPs, and each probe represents a gene, we conducted 12,360 hypothesis tests. According to the Bonferroni correction method, we set the threshold as 0.05/12360 = 4.04 e-06.

**FIGURE 4:**
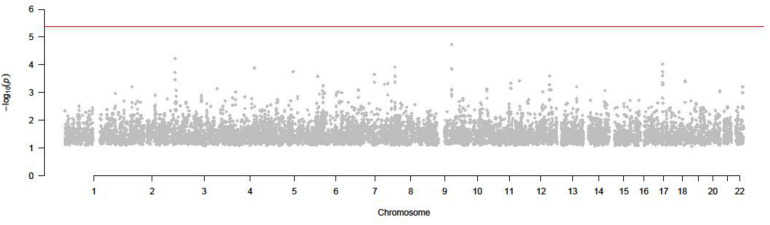
*P* value of all SNPs in SMR test (GWAS and peripheral blood eQTL).

The result of SMR by using peripheral blood eQTL and GWAS is shown in Figure 4.

As shown in Figure 4, none of SNPs passed the test and reach the significance in this test. Then we tested by thyroid eQTL dataset.

There are 17,664 probes in the thyroid eQTL dataset, so the threshold is 0.05/17664 = 2.83e-6.

**FIGURE 5:**
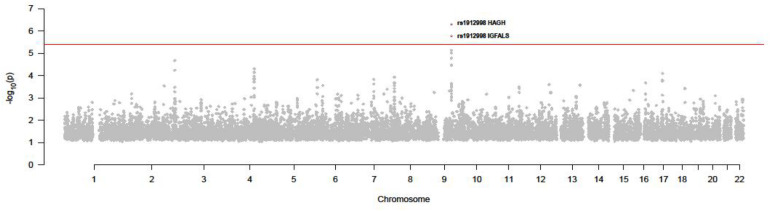
*P* value of all SNPs in SMR test (GWAS and thyroid eQTL).

As we can see in Figure 5 and Table 1, rs1912998 passed the test and regulated two gene expressions, IGFALS (*P* = 1.70E-06) and HAGH (*P* = 5.08E-07).

**TABLE 1:** Significant SNPs in SMR test.

SNP	*P*	Gene
Rs1912998	1.70E-06	IGFALS
Rs1912998	5.08E-07	HAGH

### Case Study

We explore the pathway of both IGFALS and HAGH in KEGG (Kanehisa and Goto, 2000).

IGFALS (hsa3483) is described as insulin-like growth factor binding protein acid-labile subunit, which participates in a pathway named growth hormone synthesis, secretion, and action (hsa04935) (Högler et al., 2014). The mutations caused by IGFALS are always associated with the endocrine system. According to the classification of the KEGG disease database, thyroid cancer (H00032) is a kind of malignant neoplasms of endocrine glands. In the pathway of transcriptional misregulation in cancer, which is related to the disease thyroid cancer, IGF system members are participating in the process of tumor growth and survival.

In the research of Vella et al. (2001), they found that the activation of the insulin-like growth factor (IGF) system in cancer has emerged as a key factor for tumor progression and resistance to apoptosis, and experiments validated that IGF system members were found to be locally produced in thyroid cancer.

HAGH (hsa3029) is described as hydroxyacylglutathione hydrolase, which participates in the pathways Pyruvate metabolism (hsa00620) and Metabolic pathways (hsa01100). This indicates that HAGH is associated with pyruvate kinase. Pyruvate kinase is an enzyme that catalyzes the conversion of phosphoenolpyruvate and ADP to pyruvate and ATP in glycolysis and displays a role in regulating cell metabolism (Goswami et al., 2015). TC is the most frequent endocrine tumor with a growing incidence worldwide. Recently, new emphasis has been given to the altered cellular metabolism of proliferating cancer cells which require a high amount of glucose for energy production and macromolecules biosynthesis. Also, TC displays alteration of energy metabolism orchestrated by oncogenes activation and tumor suppressors inactivation leading to abnormal proliferation. Furthermore, TC shows significant metabolic heterogeneity within the tumor microenvironment and metabolic coupling between cancer and stromal cells (Ciavardelli et al., 2017).

In the study of Liu et al. (2020), overexpression of PPAT (Phosphoribosyl pyrophosphate amidotransferase) significantly promotes tumor cell proliferation and invasion *via* activating pyruvate kinase (PK, which related to HAGH). And they identified that PPAT plays a crucial role in regulating proliferation, migration, and invasion of thyroid cancer.

### Gene Regulatory Network

We explored the interactions between IGFALS/HAGH and other genes in the String database.

**FIGURE 6:**
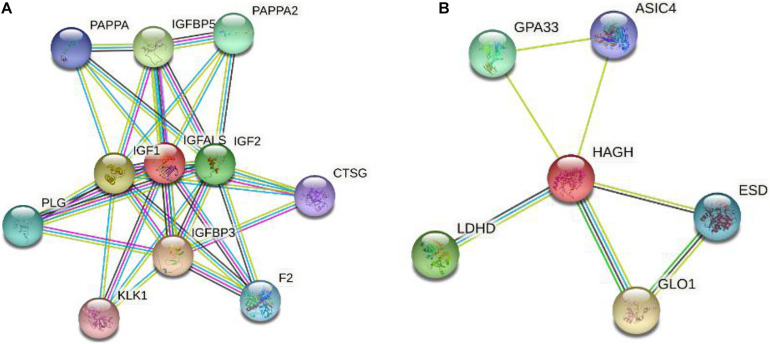
Gene interaction network of IGFALS and HAGH. **(A)** Gene interaction network of IGFALS. **(B)** Gene interaction network of HAGH.

Figure 6 only shows the interaction score above 0.7. Among these genes, several studies have reported the association between IGF1, IGFBP3, IGF2, and TC. Polat et al. (2016) enrolled seventy TC patients and 84 age-matched controls and found that IGFBP3 levels in serum were significantly higher in TC patients compared to the control group.

## Conclusion

The etiology and pathogenesis of TC are still unclear. Studies have shown that high iodine, radiation exposure, chronic lymphocytic thyroiditis, oral contraceptives, and other factors have a certain role in contributing to the incidence of TC, but not all the people exposed to these pathogenic factors will lead to the occurrence of TC. This individual difference is determined by the genetic susceptibility of the tumor. A number of research teams have used GWAS to find the genetic susceptibility locus of TC. However, the biological mechanism of these SNPs is still unknown.

In this paper, we integrated GWAS with eQTL in thyroid and blood to explore the pathogenic mechanism of susceptibility locus and find casual genes of TC. Although none of SNPs passed the SMR test in the blood eQTL dataset, the expression of the IGFALS and HAGH mediated by rs1912998 was significantly correlated with TC by using thyroid eQTL. By searching their pathway in KEGG and exploring their regulatory network, we found the functions of both IGFALS and HAGH show potential correlation with TC.

In this study, we reveal effector genes *via* GWAS with eQTL in both thyroid and blood, and provide evidence for further studies about the potential effect of these genes within TC. Although we discussed our results in multiple aspects, these conclusions still need to be further verified by biological experiments.

## Data Availability Statement

Publicly available datasets were analyzed in this study. This data can be found here. GWAS: http://ftp.ebi.ac.uk/pub/databases/gwas/summary_statistics/RashkinSR_32887889_GCST90011813 and eQTL: https://storage.googleapis.com/gtex_analysis_v8/single_tissue_qtl_data/GTEx_Analysis_v8_eQTL.tar.

## Ethics Statement

Ethical review and approval was not required for the study on human participants in accordance with the local legislation and institutional requirements. Written informed consent for participation was not required for this study in accordance with the national legislation and the institutional requirements.

## Author Contributions

FS, XG, RZ, WC, and BX carried out the GWAS with expression quantitative trait loci (eQTL) method to explore the mechanism of mutations and causal genes of thyroid cancer and participated in its design. JF, ZC, MG, YL, and ZW analyzed the data. FS, XG, and RZ wrote the manuscript. All authors read and approved the final manuscript.

## Conflict of Interest

The authors declare that the research was conducted in the absence of any commercial or financial relationships that could be construed as a potential conflict of interest.
